# EMG Indices in TMJ Disorder Patients: Insights From a Cross‐Sectional Study

**DOI:** 10.1155/ijod/9362738

**Published:** 2026-07-13

**Authors:** Junaid Ahmed, Nandita Shenoy, Anwesha Saha, Nanditha Sujir, N. Srikant, Yogesh Chhaparwal

**Affiliations:** ^1^ Department of Oral Medicine and Radiology, Manipal College of Dental Sciences Mangalore, Manipal Academy of Higher Education, Manipal, India, manipal.edu; ^2^ Department of Oral Pathology, Manipal College of Dental Sciences Mangalore, Manipal Academy of Higher Education, Manipal, India, manipal.edu; ^3^ Department of Oral Medicine and Radiology, Manipal College of Dental Sciences, Manipal, Manipal Academy of Higher Education, Manipal, India, manipal.edu

**Keywords:** electromyography, functional indices, masseter muscle, masticatory muscles, sEMG, temporal muscle, TMD

## Abstract

**Background:**

Temporomandibular disorders (TMDs) are a major public health concern, with masticatory muscle pain being the most prevalent type. Recent studies have increasingly highlighted the use of surface electromyography (sEMG) as an effective diagnostic tool for identifying muscle dysfunctions associated with TMDs. The objective of this study was to compare the electrical activity of the muscles of mastication and correlate it with chewing disability and the severity of signs and symptoms of TMDs.

**Materials and Methods:**

A total of 28 individuals (*n* = 22 women and *n* = 6 men) with the painful muscular form of TMDs were recruited alongside a numerically and gender‐matched control group—healthy, pain‐free controls. Electromyography (EMG) of the masseter and temporalis muscles was conducted on randomly recruited patients, and the symptoms of TMDs were assessed via a visual analog scale (VAS), the ProTMD multipart II questionnaire, and a chewing ability questionnaire. The results were analyzed via descriptive statistics, independent *t*‐tests, Pearson correlation coefficients, and forward stepwise linear regression analysis.

**Results:**

This study revealed significant findings regarding chewing ability and the severity of signs and symptoms associated with TMDs via the ProTMD multipart II questionnaire. The VAS scores for TMDs and mouth opening were significantly different (*p* < 0.001). The electromyographic (EMG) activity of the muscles was significant at rest for both the left masseter and temporalis muscles. When comparing the case and control groups, significant differences were observed in chewing ability, severity of TMD signs and symptoms, VAS scores, and mouth opening (*p* < 0.001). Additionally, sEMG demonstrated significant muscular activity at rest for the left masseter and left temporalis muscles.

**Conclusion:**

The sEMG protocol and questionnaire related to the chewing ability and severity of TMD are useful for evaluating patients with TMDs and their impact on the stomatognathic system.

## 1. Introduction

Temporomandibular disorders (TMDs) are quite common within the stomatognathic system, with a prevalence rate of 34% in the year 2020 and projected to reach 39% by the year 2030, according to a recent systematic review [1]. Common signs of temporomandibular dysfunction include pain in the preauricular region, soreness of the masticatory muscles, and jaw deviation during movement [2]. Pain, clicking sounds, and difficulty chewing are the most frequently reported complaints. The complex biopsychosocial factors contributing to the onset or exacerbation of TMDs have led to the consideration of multiple treatment options [3, 4].

Under normal circumstances, the stomatognathic system functions with neuromuscular synchrony, demonstrating excellent coordination among various components, such as joints and muscles [5]. However, TMDs disrupt the harmony of oromandibular structures, leading to dysfunction in the lips, tongue, mandible, and cheeks. This physiological disruption, often resulting from chronic TMDs, is collectively referred to as orofacial myofunctional disorder (OMD) [6, 7].

OMDs can manifest as difficulties in essential functions such as chewing, adversely affecting the quality of life for those affected. Individuals may experience limitations accompanied by symptoms such as jaw pain, fatigue, and jaw noise [8]. Clinical assessments often reveal reduced strength and activation of the masticatory muscles. Moreover, research indicates that TMDs can alter the preferred chewing side, resulting in the remodeling of the temporomandibular joint (TMJ) [9, 10].

Therefore, treatment must focus on restoring the functional activities of chewing, speech, and swallowing while reestablishing harmony among the orofacial structures. One of the main issues with individuals with TMDs is decreased chewing ability. Recently, a questionnaire was utilized to evaluate the chewing ability of Kurita et al. [11], which evaluated the difficulty in eating 19 different kinds of food. Healthy individuals could consume any of the food types without difficulties, whereas TMD patients had severe disability with the same foods [11].

Systematic clinical examination is a crucial initial step in diagnosing TMDs, which additional imaging techniques should complement. The research diagnostic criteria for TMDs (RDC/TMDs) and the Diagnostic criteria for TMDs (DC/TMDs) are the most commonly used standards for examining and diagnosing TMDs [12]. Clinical examinations have demonstrated sensitivity and specificity for diagnosing subluxation, disc displacement without reduction, and arthralgia. However, for other types of TMDs, specific imaging protocols are necessary to achieve better diagnostic accuracy [13].

Surface electromyography (sEMG) represents an objective and noninvasive diagnostic modality integral to evidence‑based dentistry and physiotherapy for evaluating muscular function and treatment outcomes. The technique enables quantitative assessment of bioelectrical activity generated during muscle contraction and is extensively used for the analysis of myoelectric signals of the masticatory muscles. sEMG has been applied in the evaluation of patients with TMDs, bruxism, and occlusal characteristics and during orthodontic treatment, as well as in healthy populations for baseline comparison. Furthermore, a substantial body of literature employing sEMG demonstrates the effectiveness of various therapeutic approaches in normalizing the altered masticatory muscle activity in individuals diagnosed with TMDs [14, 15].

Compared with those of healthy individuals, the masticatory muscles of symptomatic TMD patients are more asymmetrical, prone to fatigue, less efficient and coordinated, and produce lower electric potentials and bite forces [16–18]. Several questionnaires have been formulated to screen for symptoms related to TMDs; however, limited evidence correlates these symptoms with muscle activity.

On the basis of the above information, a study was conducted to compare the electric activity of the muscle and correlate it with chewing disability and the severity of the signs and symptoms of TMDs. The study hypothesis states that altered masticatory muscle activity is associated with greater chewing disability and increased severity of TMD signs and symptoms, as assessed by validated questionnaires and sEMG parameters.

## 2. Materials and Methods

The prospective case‒control study was carried out from July 2021 to August 2022 after approval from the IEC of our institution (Protocol Number 20013). The sample size was calculated on the basis of the article by Fuentes et al. [19]. Considering a 5% alpha error (*α*), 80% power (*β*), and a clinically significant difference (*d*) of 14 units, using the formula *n* = (*Z* *α*/2 + *Z* *β*)2∗2∗*σ*2/*d*2, the required sample size in each group was calculated to be 14.

Thus, 14 participants were included in the case group, and 14 age‐ and sex‐matched individuals were included in the control group. The study was conducted in three phases after informing participants about the research procedure, obtaining their informed consent, and highlighting the specific aspects of sEMG.

### 2.1. Phase 1: Diagnostic Assessment and Participant Selection

The first phase of the study involved the diagnosis of TMDs and recruitment of eligible participants using a standardized clinical examination protocol consistent with the diagnostic criteria for TMD (DC/TMD). The diagnostic process focused primarily on identifying individuals with painful muscular TMD (myalgia), with or without associated disc displacement with reduction.

The inclusion criteria were as follows: aged between 20 and 40 years, including women and men who provided consent, had four support zones in the dental arch, and had no history of surgery or injuries to the TMJ within the last 6 months.

The exclusion criteria for individuals were malocclusions (Angle Class II or III), ongoing orthodontic treatment, tumors in the head and neck region, neurological or cognitive deficits, and pregnancy.

All participants underwent a detailed clinical evaluation that included comprehensive history taking related to orofacial pain, functional limitations, and joint sounds, followed by a structured examination of the TMJ and masticatory muscles. Clinical assessment comprised palpation of the TMJ and masticatory muscles to evaluate pain and tenderness, assessment of mandibular movements for deviation or restriction, identification of joint sounds such as clicking and crepitus during jaw movements, and measurement of maximum mouth opening.

The pain intensity associated with TMD was recorded using a visual analog scale (VAS). Based on the clinical findings and DC/TMD‑consistent diagnostic criteria, participants diagnosed with painful muscular TMD (myalgia), with or without disc displacement with reduction, were included in the case group. Age‑ and sex‑matched individuals without any signs or symptoms of TMD were recruited as healthy controls.

Only participants who satisfied the diagnostic criteria, fulfilled the predefined inclusion and exclusion criteria, and provided written informed consent were enrolled for subsequent phases of the study.

The second phase of this study began with an assessment of the severity of TMD symptoms, evaluated using the “ProTMD multipart II questionnaire,” a validated tool [19] used to assess the perceived severity of TMD signs and symptoms.

The ProTMD multipart II questionnaire was used in its validated English version as originally described by De Felício et al. [20] The instrument has demonstrated good internal consistency and clinical validity, with the original validation study reporting satisfactory reliability indices (Cronbach’s *α* values indicating acceptable to high internal consistency) for the assessment of the severity of TMD signs and symptoms.

The chewing ability questionnaire was adopted from Kurita et al. [11] has been previously validated as a functional outcome measure in patients with TMDs and has demonstrated adequate reliability and construct validity for assessing masticatory disability. Both questionnaires have been widely used in TMD research and were applied in the present study without any modification.

The ProTMD multipart II questionnaire has been previously validated, demonstrating good internal consistency (Cronbach’s *α* >0.8) and clinical validity for assessing the severity of TMD signs and symptoms, as reported by De Felício et al. The chewing ability questionnaire described by Kurita et al. has also shown acceptable reliability and validity in evaluating functional impairment related to mastication in TMD patients and was used without modification in the present study.

The severity of nine TMD signs was assessed via an 11‐point numerical scale. A score of zero indicated the absence of symptoms, whereas a score of 10 represented the highest possible severity. This assessment was conducted while the subjects were waking up during activities such as chewing, speaking, and resting. The total severity score ranged from 0 to 360.

Chewing ability was assessed via a validated questionnaire [11] that evaluated the capacity to chew 10 different types of food. The scoring system was defined as follows: foods categorized as “easy to chew” were awarded 2 points, those marked as “managed to do” were given 1 point, and items identified as “difficult” to chew received 0 points. The overall score for chewing ability was calculated by summing these values.

The maximum degree of mouth opening was recorded, and TMJ pain was measured via the VAS to rate TMJ pain (no pain was rated as 0, and severe pain was scored as 10).

Muscle tenderness was assessed through palpation and classified into four categories: no palpable tenderness, minimal palpable tenderness, moderate palpable tenderness, and severe palpable tenderness. Additionally, crepitus and joint clicking were evaluated separately. Joint clicking was categorized into four levels: absent, felt, audible, and clicking. Moreover, joint crepitus was classified into three categories: no crepitus, occasional crepitus, and present crepitus All palpation assessments were performed by a single trained examiner who was calibrated prior to data collection to ensure consistency. In addition, a standardized palpation pressure of ~1 kg/cm^2^ was applied in accordance with established clinical guidelines.

Phase 3 started with electromyographic recordings and measurements according to the protocols described by other studies [16, 17]. The masseter and temporalis muscles are frequently chosen for electromyography (EMG) studies in patients with TMDs because of their primary role in mastication. Additionally, these muscles significantly change during activity and are considered a predictive marker for TMD severity.

**Figure 1:**
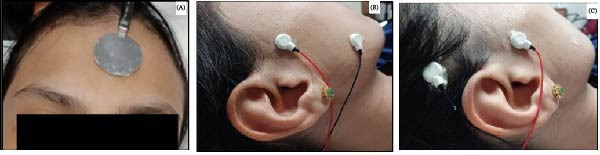
(A) Placement of a ground electrode on the forehead of a patient. (B) Placement of an active electrode (black in color) on the right masseter muscle and a reference electrode (red in color) on the skin. (C) Placement of an active electrode (black in color) on the right temporalis muscle and a reference electrode (red in color) on the skin.

A ground electrode (green) was placed on the forehead, whereas a silver/silver chloride bipolar surface electrode or an active electrode (black) was positioned over the masseter and temporalis muscles (Figure 1A,B). The placement of the surface electrodes was standardized. For the masseter muscle, the surface electrode was placed at the intersection of a line from the eye’s outer canthus to the mandible’s angle and a line from the center of the tragus to the corner of the mouth. For the temporalis muscle, the active electrode was positioned ~1 cm above the zygomatic arch and 1.5 cm posterior to the eye commissure (Figure 1C). A reference electrode (red) was placed on the skin.

**Figure 2:**
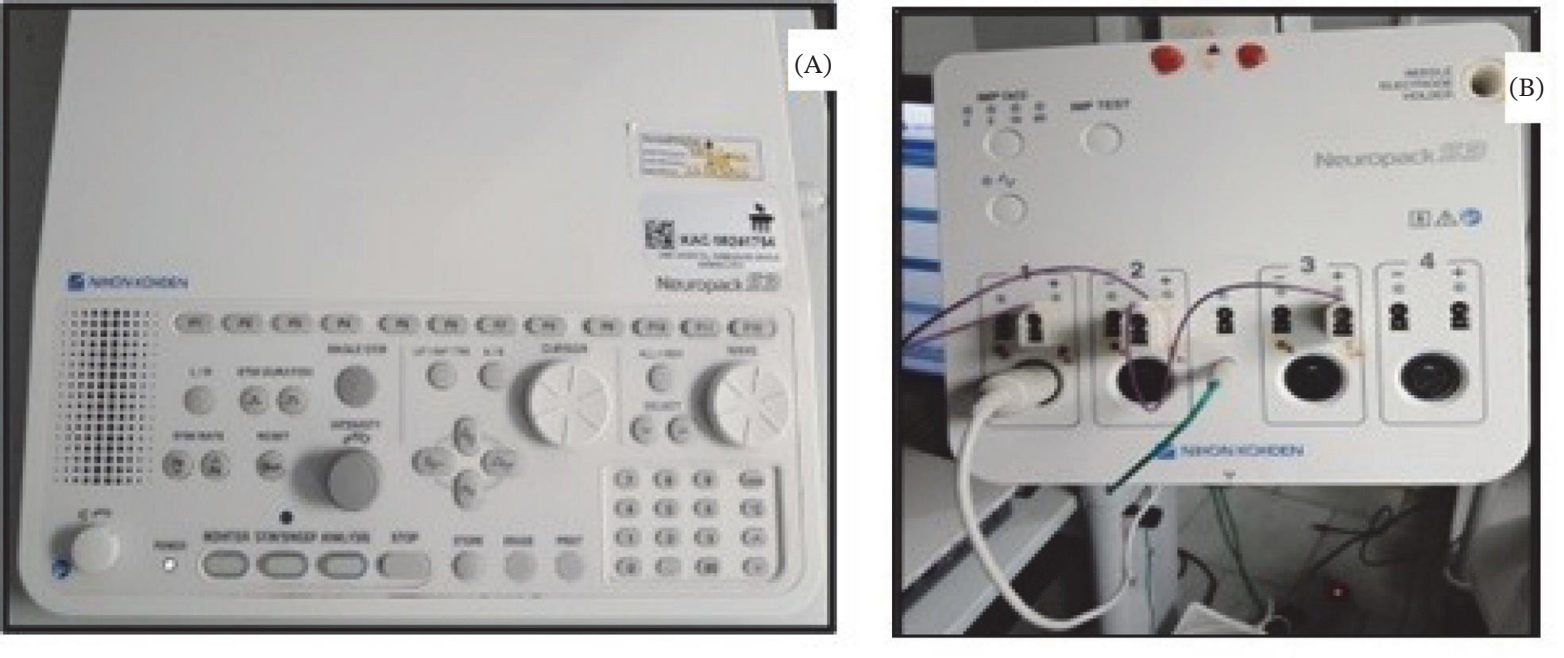
NIHON KOHDEN, NEUROPACK S3 machine used in the study.

The participants underwent sEMG testing of the bilateral masseter and temporalis muscles at rest and during maximum voluntary clenching (MVC) via the NIHON KOHDEN NEUROPACK S3 machine (Figure 2).

### 2.2. sEMG Acquisition and Signal Processing

sEMG signals were recorded using a standardized protocol for masticatory muscle assessment. The sEMG signals were acquired at a sampling frequency of 2000 Hz, which is considered adequate for capturing the frequency components of masticatory muscle activity.

The recorded signals were processed using a band‑pass filter, with a high‑pass filter set at 20 Hz to minimize movement artifacts and low‑frequency noise and a low‑pass filter set at 500 Hz to eliminate high‑frequency noise and electrical interference.

For signal normalization, electromyographic activity was normalized to MVC to allow intersubject and intermuscle comparison. MVC recordings were obtained under standardized conditions by asking participants to perform maximal clenching on two 10‐mm‐thick cotton rolls placed bilaterally between the mandibular first and second molars. Test recordings were subsequently performed without cotton rolls. The normalized sEMG values were expressed as a percentage of the MVC reference contraction.

All MVC recordings were repeated twice, with adequate rest intervals between trials to minimize muscle fatigue, and the mean value was used for analysis.

All the participants were instructed to avoid caffeine and strenuous physical activity prior to recording, attend recordings at a standardized time period, and report bruxism history and acute stress. These factors are also acknowledged as residual limitations and confounders of the study.

At rest, all the participants were instructed to relax and keep their maxillary and mandibular teeth apart, while the sEMG signals were recorded. For the MVC, both a standardized recording and a test recording were performed. The participants were asked to clench their teeth as hard as possible and hold the contraction for 5 s. For standardization, participants clenched on two 10‐mm‐thick cotton rolls placed between the mandibular first and second molars, while the test recording was performed without the cotton rolls. Each subject performed the MVC test twice, with at least a 3 min interval between tests to minimize fatigue (Table 1).

**Table 1:** Summary of study phases outlining objectives, methodology, and assessed variables.

Study phase	Objective	Methodology
Phase 1: Participant selection and symptom screening	To identify and recruit eligible TMD patients and matched healthy controls and to document baseline symptoms	Screening based on predefined inclusion and exclusion criteria; diagnosis of TMD; symptom documentation using visual analog scale (VAS)
Phase 2: Assessment of TMD severity and functional limitation	To evaluate the severity of TMD signs and symptoms and assess functional impairment related to mastication	Administration of ProTMD multipart II questionnaire; chewing ability questionnaire; clinical evaluation of mouth opening, muscle tenderness, joint clicking, and crepitus
Phase 3: Electromyographic evaluation	To assess masticatory muscle activity and compare electromyographic patterns between TMD patients and controls	Surface electromyography (sEMG) recording of bilateral masseter and temporalis muscles at rest and during maximum voluntary clenching (with and without cotton rolls)

### 2.3. Statistical Analysis

The collected data were analyzed via SPSS software version 25.0 (IBM Corp., 2017). Comparisons between the case and control groups regarding sEMG values, chewing ability, ProTMD, VAS, and mouth opening ability were conducted via independent *t*‐tests. Intragroup correlations of various parameters within the case group were assessed via Pearson’s correlation coefficient. Additionally, forward stepwise linear regression analysis was performed to predict mouth opening. A *p* value of <0.05 was considered to indicate statistical significance.

In addition to *p*‐values, effect sizes and 95% confidence intervals (CIs) were calculated to provide a more comprehensive interpretation of the results. For comparisons between groups, Cohen’s *d* was used as a measure of effect size, with values of 0.1, 0.4, and 0.9 representing small, moderate, and large effects, respectively [21]. CIs were reported where applicable to indicate the precision of the estimates. Statistical significance was set at *p* < 0.05. All analyses were performed using SPSS version 25.0 (IBM Corp., Armonk, NY, USA).

## 3. Results

A total of 14 (4 males and 10 females) cases and 14 (2 males and 12 females) controls were included in the study, with no statistically significant difference in sex distribution (*p* = 0.357) among the groups. There was no significant difference between the groups in terms of age distribution, with a *p*‐value of 0.533 (Table 2). The mean (SD) VAS score in the control group was 4.8 (± 3.8). All the subjects in the case group were diagnosed with myalgia, 9 of whom were diagnosed with disc displacement with reduction and 6 of whom had unilateral TMD.

**Table 2:** Comparison of sEMG indices, chewing ability, Pro TMD, VAS for TMD patients, and mouth opening in TMD patients and the control group.

Parameters assessed	Cases (*n* = 14)	Controls (*n* = 14)	*t*	*p*‐Value	Cohen’s *d*
	Mean ± sd	Mean ± sd			
Age	29.07 ± 10.43	27.21 ± 3.14	0.638	0.533	
Rest (RMM) (mV)	0.00127 ± 0.00003	0.00125 ± 0.00004	−1.382	0.179	0.52
Rest (LMM) (mV)	0.00132 ± 0.00004	0.00129 ± 0.00004	−2.735	0.011	1.03
Rest (RTM) (mV)	0.00183 ± 0.00002	0.00182 ± 0.00002	−1.416	0.169	0.54
Rest (LTM) (mV)	0.00193 ± 0.00004	0.00189 ± 0.00003	−2.962	0.006	1.12
RMM (without cotton) MVC (mV)	0.62 ± 0.3	0.85 ± 0.3	−2.051	0.051	−0.78
RMM (with cotton) MVC (mV)	0.69 ± 0.22	0.69 ± 0.33	−0.065	0.948	−0.02
LMM (without cotton) MVC (mV)	0.83 ± 0.42	0.84 ± 0.33	−0.049	0.961	−0.02
LMM (with cotton) MVC (mV)	0.81 ± 0.39	0.91 ± 0.42	−0.682	0.501	−0.26
RTM (without cotton) MVC (mV)	0.88 ± 0.52	0.86 ± 0.26	0.153	0.88	0.06
RTM (with cotton) MVC (mV)	0.83 ± 0.41	0.88 ± 0.38	−0.335	0.74	−0.13
LTM (without cotton) MVC (mV)	0.87 ± 0.5	0.89 ± 0.28	−0.098	0.923	−0.04
LTM (with cotton) MVC (mV)	0.78 ± 0.36	0.82 ± 0.34	−0.277	0.784	−0.1
Chewing ability (30)	14.21 ± 3.64	20 ± 0	−5.945	<0.001	−2.25
ProTMD (360)	51.14 ± 37.24	0 ± 0	5.139	<0.001	1.94
TMD (VAS)	4.86 ± 3.16	0 ± 0	5.753	<0.001	2.17
Mouth opening	40.93 ± 3.39	46.29 ± 3.02	−4.416	<0.001	−1.67

Abbreviations: LMM, left masseter muscle; LTM, left temporalis muscle; MVC, maximum voluntary clenching; RMM, right masseter muscle; RTM, right temporalis muscle.

The study revealed significant values for chewing ability and severity of TMD signs and symptoms when the ProTMD multipart II questionnaire was used. VAS scores for TMD and mouth opening (*p* < 0.001) were significantly different between the case and control groups. Muscle activity was significant at rest for the left masseter and temporalis muscles. The correlation of the results of the questionnaire with the various sEMG values did not reveal any significant results (Table 3). There were significant differences in chewing ability and mouth opening between the cases and controls, with large effect sizes observed for chewing ability (Cohen’s *d* = −2.25) and mouth opening (Cohen’s *d* = −1.67), indicating lower chewing ability and reduced mouth opening scores among the cases compared with those among the controls. Similarly, significant differences were observed for ProTMD, TMD, and VAS scores, with large effect sizes indicating higher scores among the cases than those among the controls.

**Table 3:** Correlations of the chewing ability, severity of TMD symptoms, and sEMG values among patients with TMD.

Parameters being correlated	Correlation (*r*)	*p*‐Value
Rest (RMM) (mV) and chewing ability (30)	−0.35	0.219
Rest (LMM) (mV) and chewing ability (30)	−0.054	0.854
Rest (RTM) (mV) and chewing ability (30)	−0.376	0.185
Rest (LTM) (mV) and chewing ability (30)	−0.164	0.576
RMM (without cotton) MVC (mV) and chewing ability (30)	0.088	0.764
RMM (with cotton) MVC (mV) and chewing ability (30)	0.121	0.68
LMM (without cotton) MVC (mV) and chewing ability (30)	−0.482	0.081
LMM (with cotton) MVC (mV) and chewing ability (30)	−0.456	0.101
RTM (without cotton) MVC (mV) and chewing abilility (30)	−0.184	0.528
RTM (with cotton) MVC (mV) and chewing ability (30)	−0.267	0.355
LTM (without cotton) MVC (mV) and chewing abilility (30)	−0.507	0.064
LTM (with cotton) MVC (mV) and chewing ability (30)	−0.304	0.29
Rest (RMM) (mV) and ProTMD (360)	−0.527	0.053
Rest (LMM) (mV) and ProTMD (360)	−0.452	0.105
Rest (RTM) (mV) and ProTMD (360)	0.235	0.419
Rest (LTM) (mV) and ProTMD (360)	0.284	0.325
RMM (without cotton) MVC (mV) and ProTMD (360)	0.322	0.261
RMM (with cotton) MVC (mV) and ProTMD (360)	0.164	0.576
LMM (without cotton) MVC (mV) and ProTMD (360)	0.344	0.229
LMM (with cotton) MVC (mV) and ProTMD (360)	0.184	0.528
RTM (without cotton) MVC (mV) and ProTMD (360)	0.215	0.461
RTM (with cotton) MVC (mV) and ProTMD (360)	−0.038	0.897
LTM (without cotton) MVC (mV) and ProTMD (360)	0.216	0.458
LTM (with cotton) MVC (mV) and ProTMD (360)	0.135	0.646
Chewing ability (30) and ProTMD (360)	−0.028	0.924

Abbreviations: LMM, left masseter muscle; LTM, left temporalis muscle; MVC, maximum voluntary clenching; RMM, right masseter muscle; RTM, right temporalis muscle; TMD, temporomandibular disorder.

Forward stepwise linear regression analysis (Tables 4–6) revealed that chewing ability was the single best predictor of mouth opening. Notably, mouth opening = 31.401 + 0.741 (chewing ability) ± 3.172. This has an *r* value of 0.665 (moderate correlation), and 44.2% of the mouth opening is predicted by the chewing ability. The addition of Pro TMD360 improves the correlation with mouth opening 36.180 + 0.496 (Chewing ability [30]).

**Table 4:** Forward stepwise linear regression analysis showing that chewing ability is the single best predictor of mouth opening.

Model	*R*	*R* square	Adjusted *R* square	Std. error of the estimate
1	0.665^a^	0.442	0.420	3.172
2	0.732^b^	0.535	0.498	2.952

^a^Predictors: (Constant), Chewing abilility (30).

^b^Predictors: (Constant), Chewing abilility (30), ProTMD (360).

**Table 5:** Excluded variables^a^ of the forward stepwise linear regression analysis.

Model		Beta in	*t*	Sig.	Partial correlation	Collinearity statistics
						Tolerance
1	ProTMD (360)	−0.367^b^	−2.243	0.034	−0.409	0.696
	Gender	−0.039^b^	‐0.259	0.798	−0.052	1.000
	Age	−0.192^b^	−1.295	0.207	−0.251	0.956
	Rest (RMM) (mV)	0.004^b^	0.027	0.979	0.005	0.881
	Rest (LMM) (mV)	0.033^b^	0.205	0.839	0.041	0.856
	Rest (RTM) (mV)	0.156^b^	0.988	0.333	0.194	0.862
	Rest (LTM) (mV)	−0.208^b^	−1.280	0.212	−0.248	0.795
2	Gender	−0.006^c^	−0.044	0.965	−0.009	0.988
	Age	−0.154^c^	−1.100	0.282	−0.219	0.940
	Rest (RMM) (mV)	−0.107^c^	−0.695	0.494	−0.141	0.796
	Rest (LMM) (mV)	0.005^c^	0.032	0.975	0.007	0.849
	Rest (RTM) (mV)	0.201^c^	1.383	0.179	0.272	0.848
	Rest (LTM) (mV)	−0.108^c^	−0.663	0.514	−0.134	0.712

^a^Dependent variable: mouth opening.

^b^Predictors in the model: (Constant), Chewing abilility (30).

^c^Predictors in the model: (Constant), Chewing abilility (30), ProTMD (360).

**Table 6:** Coefficients^a^ of the forward stepwise linear regression analysis.

Model		Unstandardized coefficients		Standardized coefficients	*t*	*p*‐Value	95.0% confidence interval for *B*	
		*B*	Std. Error	Beta			Lower bound	Upper bound
1	(Constant)	31.401	2.757		11.390	0.000	25.734	37.068
	Chewing abilility (30)	0.714	0.157	0.665	4.536	<0.001	0.390	1.037
2	(Constant)	36.180	3.335		10.849	0.000	29.312	43.048
	Chewing abilility (30)	0.496	0.175	0.462	2.829	0.009	0.135	0.858
	ProTMD (360)	−0.042	0.019	−0.367	−2.243	0.034	−0.080	−0.003

^a^Dependent variable: mouth opening.

ProTMD (360) ± 2.952, with an *r* value of 0.732 and a 53.5% correlation. Chewing ability was significantly lower in the TMD group compared to controls (*p* < 0.001), with a large effect size, indicating a clinically meaningful difference.

## 4. Discussion

sEMG of the masticatory muscles is a type of quantitative evaluation used in the assessment of patients suffering from TMDs [16]. sEMG was performed using surface electrodes. It can explore a large area of muscle and is a noninvasive method to assess changes in electrical muscular activity at rest and at maximum muscular contraction [19]. The symmetry between the right and left muscle pairs, as well as the balanced activation of the contralateral masseter and temporal muscles, are characteristics of the relationship between the elevator muscles and their actions on the stomatognathic system [1]. The impact of occlusion on neuromuscular activity, anatomical variances, and physiological and psychological status, as well as on technical variables such as electrode position, can be shown by normal EMG data [22]. Compared with pain‐free individuals, TMD patients with pain exhibited reduced masticatory muscle electrical potentials during the MVC. There was also a decrease in muscular force during clenching, which may be due to the intricate interactions between the bite force and muscle activation [23]. Numerous muscles are involved in mastication, namely, the temporalis, masseter, pterygoid, digastric, mylohyoid, and geniohyoid muscles. The masseter and temporalis muscles have been extensively studied because of their accessibility, size, superficial location, and prominence [24].

In the present study, the difference in muscle activity measured by sEMG between the cases and the control group was significant at rest for the left masseter and left temporalis muscles, with higher activity observed in the cases than in the control group. Our study is supported by the results of a study conducted by Rodrigues et al. [25], Lauriti et al. [26], and Sójka et al. [27]. These studies concluded that at rest, there was a statistically significant difference between the TMD group and the control group, with the TMD group showing greater myoelectric activity. Notably, in TMD patients, there is increased muscle pain and hyperactivity of the masticatory muscles, which may cause muscular fatigue and pain. Muscle activity, when the mandible is at rest, relies on the lengthening reflex and is maintained by muscle tonicity; therefore, it is essential to observe the sEMG activity of the masseter and temporalis muscles at rest [21, 22].

The increased left‑sided muscle activity observed at rest may reflect a neuromuscular imbalance associated with painful muscular TMD. Such asymmetry has been widely reported in sEMG studies and is thought to result from pain‑related motor adaptations, habitual functional patterns, or unilateral loading of the masticatory system. Resting sEMG activity appears to be more sensitive to these alterations than maximal clenching tasks, which may explain the side‑specific findings in the present study. Studies have also revealed that there is marked asymmetry and uncoordinated contractile muscle activity in the masseter and temporalis muscles, which could result in differences in sEMG bilaterally [28–30].

Our study also showed results similar to those of previous studies. We found that the sEMG activity of the masseter and temporalis muscles in the case and control groups during MVC was not significantly different. Tests were conducted both with and without cotton. The standardized sEMG recording when clenching on 10‐mm‐thick cotton rolls and intercuspation (without cotton) was used as a static isometric test for MVC, as described by Ferrario et al. [16]. Our findings align with those of Fuentes et al. [19], who reported no significant differences in EMG activity for the masticatory muscles between TMD patients and control individuals. Similarly, Rodrigues et al. [25] reported no significant relationship between the muscular activity of sEMG in the case and control groups during MVC.

Patients with TMD often exhibit altered pain perception and differences in pain modulation processes, which contribute to the intensity of pain. TMD‐related pain varies widely, ranging from continuous to intermittent, with periods of exacerbation and remission [31].

The VAS scores in this study were comparable to those reported in a study by Sójka et al. [27]. Chewing ability was significantly greater in the control group. In patients with TMDs, chewing ability is affected by alterations in the neuromuscular relationship of the jaw. The motor nuclei located in the brainstem play crucial roles in influencing chewing patterns, rhythm, and intensity. Efferent activities from the relevant motor neurons can trigger cyclic contractions of the muscles responsible for chewing. Any significant disruption in this process can lead to a decline in the quality of life [32]. Additionally, as expected, there was a significant difference in mouth opening between the case and control groups because of the contracture of the masticatory muscles [33]. According to a study by Kurita et al. [11], the chewing ability score is correlated with TMJ pain and the mouth opening capacity. There was no correlation between the sEMG values and the perceived severity of symptoms or chewing ability among the subjects in the case group. In contrast, the study by De Felicio et al. [1] demonstrated a direct correlation between EMG characteristics and TMD severity. However, long‐standing arthrogenous TMDs have shown no significant difference in total muscle activity [17]. The variation in the results could be related to differences in patient perception of pain and differences in changes in electric muscle activity depending on the type of TMD and its duration. Additionally, the results also indicate that the chewing ability questionnaire and Pro TMD360 questionnaire can be utilized in combination with screening patients for reduced mouth opening. The masseter and medial pterygoid muscles are the primary elevators of the mandible and play crucial roles in jaw closure. In individuals with TMD, these muscles frequently display increased electromyographic (EMG) activity. This increase in activity can cause muscle hyperactivity or spasms, which can limit the jaw movement and lead to reduced mouth opening [34]. Additionally, the results suggest that the ProTMD multiparty II questionnaire and the chewing ability questionnaire can be used together to screen for patients with TMD and reduced mouth opening. Importantly, the observed statistically significant differences were accompanied by moderate to large effect sizes, suggesting that the findings are not only statistically significant but also clinically relevant. Reporting effect sizes alongside *p*‐values provides a more meaningful interpretation of the magnitude of group differences.

The study included a greater proportion of female participants than that of male participants. The null hypothesis suggests that there is no correlation between muscle electrical activity and either chewing disability or the severity of TMD signs and symptoms. Although TMDs are more prevalent in females, employing additional sampling techniques could help ensure greater male representation in future studies. Moreover, future research would benefit from focusing on a larger sample of specific TMD subtypes, such as disc displacement, myalgias, and osteoarthritis. This approach could provide deeper insights into the muscle activity associated with different forms of TMD.

This study highlights the clinical relevance of sEMG as an objective, noninvasive tool for the assessment of masticatory muscle activity in individuals with TMDs. The findings demonstrate that sEMG is sensitive to detecting alterations in bioelectrical muscle activity and is valuable in evaluating functional changes associated with therapeutic interventions. The observed improvements in muscle activity patterns following treatment underscore the effectiveness of the therapeutic approach employed and support the role of sEMG in monitoring neuromuscular adaptation.

The integration of sEMG into evidence‑based dental and physiotherapeutic practice enhances diagnostic accuracy and provides quantifiable outcomes that complement clinical examination. By offering reproducible and objective measurements, sEMG aids in understanding the functional status of the masticatory system and contributes to informed clinical decision‑making.

Despite its strengths, the study acknowledges certain limitations, including the sample size and study duration, which may influence the generalizability of the results. Future research with larger, diverse populations and long‑term follow‑up is recommended to further validate these findings and to establish standardized sEMG protocols for routine clinical use.

## 5. Conclusion

sEMG serves as a reliable and effective adjunctive diagnostic and evaluative tool in the management of TMDs, supporting its continued application in both clinical practice and research aimed at improving patient‑centered outcomes.

Future studies with larger sample sizes, a focus on specific TMJ disorders, and long‐term follow‐up can enhance our understanding of TMDs. Additionally, changes in treatment protocols may be required to achieve favorable results in managing these complex, multifactorial cases.

## Author Contributions

**Junaid Ahmed:** data collection, formal analysis, writing – original draft. **Nandita Shenoy:** conceptualization, methodology, supervision, writing – review and editing. **Anwesha Saha:** data collection, validation, writing – review and editing. **Nanditha Sujir:** literature review, data interpretation, writing – review and editing. **N. Srikant:** statistical analysis, data curation, writing – review and editing. **Yogesh Chhaparwal:** visualization, formatting, writing – review and editing.

## Funding

The authors received no specific funding for this work.

## Disclosure

All authors have read and approved the final version of the manuscript and agree to be accountable for all aspects of the work. All content generated or assisted by AI tools was thoroughly reviewed, verified, and approved by the authors to ensure accuracy, originality, and compliance with journal standards.

## Consent

We confirm that written informed consent was obtained from all participants included in the study. This includes the consent for the use of clinical data and any identifiable images.

## Conflicts of Interest

The authors declare no conflicts of interest.

## Data Availability

The data that support the findings of this study are available upon request from the corresponding author. The data are not publicly available due to privacy or ethical restrictions.
